# *tert*-Butyl 2-methyl-2-(4-methyl­benzo­yl)propanoate

**DOI:** 10.1107/S1600536810003144

**Published:** 2010-01-30

**Authors:** Graham B. Gould, Brock G. Jackman, Marshall W. Logue, Rudy L. Luck, Louis R. Pignotti, Adrian R. Smith, Nicholas M. White

**Affiliations:** a Department of Chemistry, 1400 Townsend Drive, Michigan Technological University, Houghton, MI 49931, USA

## Abstract

The title compound, C_16_H_22_O_3_, is bent with a dihedral angle of 75.3 (1)° between the mean planes of the benzene ring and a group encompassing the ester functionality (O=C—O—C). In the crystal, the mol­ecules are linked into infinite chains held together by weak C—H⋯O hydrogen-bonded inter­actions between an H atom on the benzene ring of one mol­ecule and an O atom on the ketone functionality of an adjacent mol­ecule. The chains are arranged with neighbouring *tert*-butyl and dimethyl groups on adjacent chains exhibiting hydro­phobic stacking, with short C—H⋯H—C contacts (2.37 Å) between adjacent chains

## Related literature

For the synthesis, spectroscopic characterization and reactivity of the title compound, see: Logue (1974 ▶); Logue *et al.* (1975 ▶). For related structures, see: Crosse *et al.* (2010*a* ▶,*b* ▶; Logue *et al.* (2010 ▶). For the syntheses and characterization of structurally similar indanone-derived β-keto ester derivatives, see: Mouri *et al.* (2009 ▶); Noritake *et al.* (2008 ▶); Rigby & Dixon (2008 ▶). For weak hydrogen-bonded inter­actions, see: Karle *et al.* (2009 ▶). For H⋯H inter­actions, see: Alkorta *et al.* (2008 ▶). 
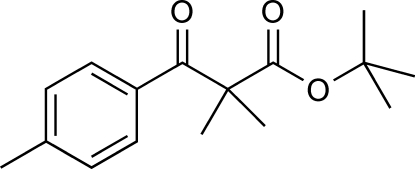


## Experimental

### 

#### Crystal data

C_16_H_22_O_3_
*M* *_r_* = 262.34
Orthorhombic, 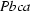

*a* = 8.605 (3) Å
*b* = 11.659 (3) Å
*c* = 31.347 (9) Å
*V* = 3144.9 (16) Å^3^
*Z* = 8
Mo *K*α radiation
μ = 0.08 mm^−1^
*T* = 291 K
0.50 × 0.30 × 0.10 mm

#### Data collection

Enraf–Nonius TurboCAD-4 diffractometer
Absorption correction: ψ scan (North *et al.*, 1968 ▶) *T*_min_ = 0.969, *T*_max_ = 0.988
4411 measured reflections
2758 independent reflections
1334 reflections with *I* > 2σ(*I*)
*R*_int_ = 0.027
3 standard reflections every 166 min  intensity decay: 2%

#### Refinement

*R*[*F* ^2^ > 2σ(*F* ^2^)] = 0.049
*wR*(*F* ^2^) = 0.134
*S* = 1.01
2758 reflections
178 parameters
H-atom parameters constrained
Δρ_max_ = 0.14 e Å^−3^
Δρ_min_ = −0.13 e Å^−3^

### 

Data collection: *CAD-4 EXPRESS* (Enraf–Nonius, 1994 ▶); cell refinement: *CAD-4 EXPRESS*; data reduction: *XCAD4* (Harms & Wocadlo, 1995 ▶); program(s) used to solve structure: *SIR2004* (Burla *et al.*, 2005 ▶); program(s) used to refine structure: *SHELXL97* (Sheldrick, 2008 ▶); molecular graphics: *ORTEP-3 for Windows* (Farrugia, 1997 ▶) and *Mercury* (Macrae *et al.*, 2008 ▶); software used to prepare material for publication: *WinGX* (Farrugia, 1999 ▶) and *publCIF* (Westrip, 2010 ▶).

## Supplementary Material

Crystal structure: contains datablocks global, I. DOI: 10.1107/S1600536810003144/zl2266sup1.cif

Structure factors: contains datablocks I. DOI: 10.1107/S1600536810003144/zl2266Isup2.hkl

Additional supplementary materials:  crystallographic information; 3D view; checkCIF report

## Figures and Tables

**Table 1:** Hydrogen-bond geometry (Å, °)

*D*—H⋯*A*	*D*—H	H⋯*A*	*D*⋯*A*	*D*—H⋯*A*
C3—H3⋯O9^i^	0.93	2.71	3.407 (3)	133

Symmetry code: (i) 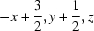.

**Fig. 1.**
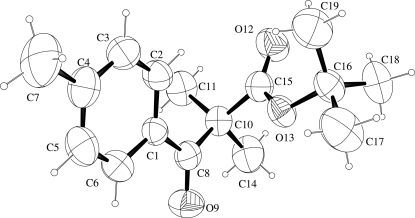
ORTEP-3 (Farrugia, 1997) drawing of the title compound with displacement ellipsoids drawn at the 50% probability level.

**Fig. 2.**
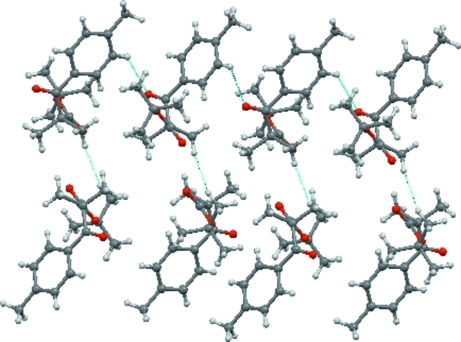
A Mercury (Macrae et al., 2008) illustration of the title compound depicting the H-bonded linkages and the C—H···H—C interactions between the chains displayed along the horizontal middle of the diagram both using dashed blue lines.

## supplementary crystallographic information

### Comment

Treatment of 2,2-disubstituted *t*-butyl beta-keto esters with trifluoroacetic acid at room temperature quantitatively generates the corresponding 2,2-disubstituted β-keto acids, which were used to probe the nature of the transition state for the thermal decarboxylation of β-keto acids (Logue *et al.*, 1975).  Structurally similar indanone-derived β-keto ester derivatives have been prepared recently (Mouri *et al.*, 2009; Noritake *et al.*, 2008; Rigby & Dixon, 2008). The directing nature of weak C—H···O H-bonds has been noted to be of importance to afford the three dimensional structure observed in these kinds of molecules (Karle *et al.*, 2009).

In this contribution we present the solid state structure of one such 2,2-disubstituted β-keto acid, i.e. the title compound being the tolyl derivative. This is the second paper in a series of four dealing with substituted derivatives (H–, CH_3_– (this paper), Cl- and NO_2_– on the *para*-position of the phenyl ring) of the title compound. A more detailed comparison of all four substitution compounds will be given in the fourth paper of this series (Crosse *et al.*, 2010*a*).

The molecule, Fig. 1, displays a bent geometry with a dihedral angle between the phenyl ring and a plane composed of the ester functionality of 75.3 (1)°. Molecules are linked by C—H···O weak hydrogen bonds generating infinite chains parallel to the *b* axis as shown in Fig. 2. The aromatic rings are not involved in intercalation of stacking interactions either within or between the chains. The chains are arranged with neighbouring *t*-butyl and dimethyl groups on adjacent chains exhibiting hydrophobic stacking with short C—H···H—C contacts between adjacent chains, Fig. 2 (Alkorta *et al.*, 2008).

### Experimental

Crystals of the material were synthesized as reported earlier and were grown by evaporation of a solution in hexane (Logue, 1974). IR (neat, cm^-1^): 3003 (w, C—H), 2974, 1734 (v.s., ester C=O), 1671 (v.s., ketone C=O) 1608 (m, C—C), 1455 (*m*), 1386 (*m*), 1366 (*s*), 1273 (s, alkyl methyl C—H), 1247 (*s*), 1130 (v.s., ester C—O), 986 (*s*), 921 (*m*), 836 (s, C—H bend), 740 (*s*). ^1^H NMR (CDCl_3_) δ; 1.28 (s, 9*H*), 1.47 (s, 6*H*), 2.37 (s, 3*H*), 7.19 (d, 2*H*, J=8.0 Hz), 7.76 (d, 2*H*, J=8.8 Hz). ^13^C NMR (CDCl_3_) δ; 21.7, 24.1, 27.8, 54.1, 81.8, 129.2, 132.9, 143.5, 174.4, 198.9.

### Refinement

All H atoms were placed at calculated positions, with C—H = 0.93 Å (aromatic) or 0.96 Å (methyl) and refined using a riding model with *U*_iso_(H) constrained to be 1.5 *U*_eq_(C) for methyl groups and 1.2 *U*_eq_(C) for all other C atoms. The quality of the data as reflected by only 48% of the reflections observed, large ADP's and inaccurate C—C bond lengths is low. The data had been collected on a 30 year old single point detector instrument not equipped with a low temperature device as part of a class project with undergraduate students. Due to the time constraints imposed by the class schedule a maximum exposure time of 60 s had to be alloted for measuring each reflection.

There are close contacts (*i.e.*, <2.4 Å, (Alkorta *et al.*, 2008)) between an H atom on C11 and one on the C18 atom of an adjacent molecule, Fig. 2. These contacts remain present irrespective of if all the H atoms are refined freely (which generates reasonable parameters) or if they are refined generated either with the AFIX 33 or AFIX 137 constraints (used here) as implemented in the Shelxtl software (Sheldrick, 2008).

### Crystal data

**Table:**

C_16_H_22_O_3_	*F*(000) = 1136
*M**_r_* = 262.34	*D*_x_ = 1.108 Mg m^−^^3^
Orthorhombic, *P**b**c**a*	Mo *K*α radiation, λ = 0.71073 Å
Hall symbol: -P 2ac 2ab	Cell parameters from 25 reflections
*a* = 8.605 (3) Å	θ = 10–15°
*b* = 11.659 (3) Å	µ = 0.08 mm^−^^1^
*c* = 31.347 (9) Å	*T* = 291 K
*V* = 3144.9 (16) Å^3^	Prism, colourless
*Z* = 8	0.50 × 0.30 × 0.10 mm

### Data collection

**Table:**

Enraf–Nonius TurboCAD-4 diffractometer	1334 reflections with *I* > 2σ(*I*)
Radiation source: Enraf Nonius FR590	*R*_int_ = 0.027
graphite	θ_max_ = 25.0°, θ_min_ = 1.3°
non–profiled ω scans	*h* = 0→10
Absorption correction: ψ scan (North *et al.*, 1968)	*k* = 0→13
*T*_min_ = 0.969, *T*_max_ = 0.988	*l* = −33→37
4411 measured reflections	3 standard reflections every 166 min
2758 independent reflections	intensity decay: 2%

### Refinement

**Table:**

Refinement on *F*^2^	Secondary atom site location: difference Fourier map
Least-squares matrix: full	Hydrogen site location: inferred from neighbouring sites
*R*[*F*^2^ > 2σ(*F*^2^)] = 0.049	H-atom parameters constrained
*wR*(*F*^2^) = 0.134	*w* = 1/[σ^2^(*F*_o_^2^) + (0.0573*P*)^2^ + 0.2581*P*] where *P* = (*F*_o_^2^ + 2*F*_c_^2^)/3
*S* = 1.01	(Δ/σ)_max_ < 0.001
2758 reflections	Δρ_max_ = 0.14 e Å^−^^3^
178 parameters	Δρ_min_ = −0.13 e Å^−^^3^
0 restraints	Extinction correction: *SHELXL97* (Sheldrick, 2008)
Primary atom site location: structure-invariant direct methods	Extinction coefficient: 0.0033 (5)

### Special details

**Table:**

Experimental. Number of psi-scan sets used was 6. Theta correction was applied. Averaged transmission function was used. No Fourier smoothing was applied.
Geometry. All s.u.'s (except the s.u. in the dihedral angle between two l.s. planes) are estimated using the full covariance matrix. The cell s.u.'s are taken into account individually in the estimation of s.u.'s in distances, angles and torsion angles; correlations between s.u.'s in cell parameters are only used when they are defined by crystal symmetry. An approximate (isotropic) treatment of cell s.u.'s is used for estimating s.u.'s involving l.s. planes.
Refinement. Refinement of *F*^2^ against ALL reflections. The weighted *R*-factor *wR* and goodness of fit *S* are based on *F*^2^, conventional *R*-factors *R* are based on *F*, with *F* set to zero for negative *F*^2^. The threshold expression of *F*^2^ > 2σ(*F*^2^) is used only for calculating *R*-factors(gt) *etc*. and is not relevant to the choice of reflections for refinement. *R*-factors based on *F*^2^ are statistically about twice as large as those based on *F*, and *R*- factors based on ALL data will be even larger.

### Fractional atomic coordinates and isotropic or equivalent isotropic displacement parameters (Å^2^)

**Table:**

	*x*	*y*	*z*	*U*_iso_*/*U*_eq_	
C1	0.6338 (3)	0.2128 (2)	0.65195 (7)	0.0474 (6)	
C2	0.7278 (3)	0.3098 (2)	0.65125 (7)	0.0558 (7)	
H2	0.7806	0.3292	0.6264	0.067*	
C3	0.7434 (3)	0.3773 (2)	0.68702 (8)	0.0647 (8)	
H3	0.8068	0.4417	0.6857	0.078*	
C4	0.6681 (4)	0.3524 (3)	0.72464 (9)	0.0701 (8)	
C5	0.5731 (4)	0.2569 (3)	0.72513 (9)	0.0789 (9)	
H5	0.5193	0.2385	0.7499	0.095*	
C6	0.5566 (3)	0.1886 (2)	0.68967 (8)	0.0655 (8)	
H6	0.4922	0.1247	0.691	0.079*	
C7	0.6872 (5)	0.4275 (3)	0.76352 (9)	0.1102 (13)	
H7A	0.5982	0.419	0.7817	0.165*	
H7B	0.6963	0.5062	0.7548	0.165*	
H7C	0.7791	0.4052	0.7787	0.165*	
C8	0.6120 (3)	0.1326 (2)	0.61524 (8)	0.0517 (7)	
O9	0.5392 (2)	0.04387 (16)	0.62062 (6)	0.0725 (6)	
C10	0.6724 (3)	0.1633 (2)	0.57086 (8)	0.0527 (7)	
C11	0.5841 (3)	0.2689 (2)	0.55401 (8)	0.0724 (9)	
H111	0.5999	0.3323	0.573	0.109*	
H112	0.4752	0.2516	0.5524	0.109*	
H113	0.6221	0.2884	0.5262	0.109*	
O12	0.9115 (2)	0.25100 (18)	0.54691 (6)	0.0790 (6)	
O13	0.91700 (19)	0.11790 (14)	0.59947 (5)	0.0560 (5)	
C14	0.6468 (4)	0.0623 (3)	0.54016 (8)	0.0826 (9)	
H14A	0.6857	0.082	0.5124	0.124*	
H14B	0.5378	0.0456	0.5383	0.124*	
H14C	0.701	−0.004	0.5506	0.124*	
C15	0.8467 (3)	0.1858 (2)	0.57099 (8)	0.0557 (7)	
C16	1.0892 (3)	0.1174 (2)	0.60458 (8)	0.0608 (7)	
C17	1.1112 (4)	0.0345 (3)	0.64115 (10)	0.1023 (12)	
H17A	1.0663	−0.0383	0.6338	0.153*	
H17B	1.0611	0.0641	0.6662	0.153*	
H17C	1.2202	0.0249	0.6467	0.153*	
C18	1.1633 (4)	0.0715 (3)	0.56456 (10)	0.0879 (10)	
H181	1.2716	0.0574	0.5697	0.132*	
H182	1.1522	0.1266	0.542	0.132*	
H183	1.1132	0.0011	0.5565	0.132*	
C19	1.1454 (4)	0.2360 (3)	0.61636 (11)	0.0997 (11)	
H19A	1.0827	0.2658	0.6392	0.15*	
H19B	1.1372	0.2856	0.592	0.15*	
H19C	1.2518	0.2321	0.6254	0.15*	

### Atomic displacement parameters (Å^2^)

**Table:**

	*U*^11^	*U*^22^	*U*^33^	*U*^12^	*U*^13^	*U*^23^
C1	0.0430 (15)	0.0492 (15)	0.0498 (14)	0.0041 (13)	0.0019 (12)	0.0026 (12)
C2	0.0596 (18)	0.0602 (16)	0.0476 (14)	−0.0026 (15)	0.0060 (14)	0.0001 (13)
C3	0.0670 (19)	0.0641 (17)	0.0630 (16)	−0.0065 (16)	−0.0012 (17)	−0.0118 (14)
C4	0.076 (2)	0.077 (2)	0.0570 (18)	0.0119 (18)	−0.0004 (16)	−0.0141 (16)
C5	0.093 (2)	0.091 (2)	0.0528 (16)	0.003 (2)	0.0254 (17)	−0.0023 (17)
C6	0.0686 (19)	0.0664 (18)	0.0615 (17)	−0.0029 (16)	0.0132 (15)	0.0052 (15)
C7	0.130 (3)	0.124 (3)	0.077 (2)	0.007 (3)	0.006 (2)	−0.043 (2)
C8	0.0408 (17)	0.0548 (16)	0.0596 (16)	0.0055 (14)	−0.0066 (12)	0.0018 (14)
O9	0.0761 (15)	0.0639 (12)	0.0774 (12)	−0.0182 (11)	−0.0017 (11)	−0.0012 (10)
C10	0.0520 (17)	0.0610 (17)	0.0451 (14)	0.0039 (14)	−0.0048 (13)	−0.0042 (13)
C11	0.069 (2)	0.088 (2)	0.0601 (17)	0.0141 (17)	−0.0107 (15)	0.0107 (15)
O12	0.0748 (14)	0.0883 (14)	0.0741 (12)	0.0026 (12)	0.0128 (11)	0.0238 (12)
O13	0.0419 (11)	0.0711 (12)	0.0551 (10)	0.0015 (9)	−0.0019 (9)	0.0054 (9)
C14	0.077 (2)	0.096 (2)	0.074 (2)	−0.0039 (19)	−0.0078 (16)	−0.0319 (17)
C15	0.0585 (19)	0.0615 (17)	0.0471 (15)	0.0036 (16)	0.0019 (15)	−0.0046 (15)
C16	0.0424 (17)	0.0749 (19)	0.0651 (17)	−0.0023 (15)	−0.0056 (13)	0.0028 (15)
C17	0.067 (2)	0.139 (3)	0.101 (2)	0.002 (2)	−0.0184 (19)	0.042 (2)
C18	0.062 (2)	0.113 (3)	0.089 (2)	0.013 (2)	0.0140 (17)	−0.005 (2)
C19	0.068 (2)	0.103 (3)	0.128 (3)	−0.013 (2)	−0.017 (2)	−0.022 (2)

### Geometric parameters (Å, °)

**Table:**

C1—C6	1.385 (3)	C11—H112	0.96
C1—C2	1.390 (3)	C11—H113	0.96
C1—C8	1.495 (3)	O12—C15	1.207 (3)
C2—C3	1.376 (3)	O13—C15	1.338 (3)
C2—H2	0.93	O13—C16	1.491 (3)
C3—C4	1.376 (4)	C14—H14A	0.96
C3—H3	0.93	C14—H14B	0.96
C4—C5	1.382 (4)	C14—H14C	0.96
C4—C7	1.509 (4)	C16—C18	1.505 (4)
C5—C6	1.375 (4)	C16—C19	1.511 (4)
C5—H5	0.93	C16—C17	1.511 (4)
C6—H6	0.93	C17—H17A	0.96
C7—H7A	0.96	C17—H17B	0.96
C7—H7B	0.96	C17—H17C	0.96
C7—H7C	0.96	C18—H181	0.96
C8—O9	1.221 (3)	C18—H182	0.96
C8—C10	1.528 (3)	C18—H183	0.96
C10—C15	1.523 (4)	C19—H19A	0.96
C10—C14	1.537 (3)	C19—H19B	0.96
C10—C11	1.540 (3)	C19—H19C	0.96
C11—H111	0.96		
			
C6—C1—C2	117.3 (2)	H111—C11—H113	109.5
C6—C1—C8	118.0 (2)	H112—C11—H113	109.5
C2—C1—C8	124.7 (2)	C15—O13—C16	121.6 (2)
C3—C2—C1	120.6 (2)	C10—C14—H14A	109.5
C3—C2—H2	119.7	C10—C14—H14B	109.5
C1—C2—H2	119.7	H14A—C14—H14B	109.5
C4—C3—C2	122.2 (3)	C10—C14—H14C	109.5
C4—C3—H3	118.9	H14A—C14—H14C	109.5
C2—C3—H3	118.9	H14B—C14—H14C	109.5
C3—C4—C5	117.2 (3)	O12—C15—O13	125.5 (3)
C3—C4—C7	121.2 (3)	O12—C15—C10	124.2 (3)
C5—C4—C7	121.5 (3)	O13—C15—C10	110.2 (2)
C6—C5—C4	121.3 (3)	O13—C16—C18	109.4 (2)
C6—C5—H5	119.4	O13—C16—C19	109.9 (2)
C4—C5—H5	119.4	C18—C16—C19	113.2 (3)
C5—C6—C1	121.5 (3)	O13—C16—C17	102.0 (2)
C5—C6—H6	119.3	C18—C16—C17	110.6 (3)
C1—C6—H6	119.3	C19—C16—C17	111.1 (3)
C4—C7—H7A	109.5	C16—C17—H17A	109.5
C4—C7—H7B	109.5	C16—C17—H17B	109.5
H7A—C7—H7B	109.5	H17A—C17—H17B	109.5
C4—C7—H7C	109.5	C16—C17—H17C	109.5
H7A—C7—H7C	109.5	H17A—C17—H17C	109.5
H7B—C7—H7C	109.5	H17B—C17—H17C	109.5
O9—C8—C1	119.2 (2)	C16—C18—H181	109.5
O9—C8—C10	119.9 (2)	C16—C18—H182	109.5
C1—C8—C10	120.8 (2)	H181—C18—H182	109.5
C15—C10—C8	111.9 (2)	C16—C18—H183	109.5
C15—C10—C14	106.0 (2)	H181—C18—H183	109.5
C8—C10—C14	110.0 (2)	H182—C18—H183	109.5
C15—C10—C11	110.4 (2)	C16—C19—H19A	109.5
C8—C10—C11	109.4 (2)	C16—C19—H19B	109.5
C14—C10—C11	109.1 (2)	H19A—C19—H19B	109.5
C10—C11—H111	109.5	C16—C19—H19C	109.5
C10—C11—H112	109.5	H19A—C19—H19C	109.5
H111—C11—H112	109.5	H19B—C19—H19C	109.5
C10—C11—H113	109.5		

### Hydrogen-bond geometry (Å, °)

**Table:**

*D*—H···*A*	*D*—H	H···*A*	*D*···*A*	*D*—H···*A*
C3—H3···O9^i^	0.93	2.71	3.407 (3)	133

Symmetry codes: (i) −*x*+3/2, *y*+1/2, *z*.
